# The choroid plexus- cerebrospinal fluid axis as a lifespan regulator of neural stem cells and circuit plasticity

**DOI:** 10.3389/fncir.2026.1818927

**Published:** 2026-04-22

**Authors:** Kelren S. Rodrigues, Rie Yamashita, Sayako Katada

**Affiliations:** 1 Department of Stem Cell Biology and Medicine, Graduate School of Medical Sciences, Kyushu University, Higashi-ku, Fukuoka, Japan; 2 Center for iPS Cell Research and Application (CiRA), Kyoto University, Sakyo-ku, Kyoto, Japan

**Keywords:** cerebrospinal fluid, choroid plexus, circuit plasticity, homeostasis, neural stem cell

## Abstract

The choroid plexus-cerebrospinal fluid axis (ChP-CSF) functions as a dynamic signaling system that coordinates neural stem cell (NSC) behavior and neural circuit plasticity across the lifespan. Beyond its classical roles in cushioning the brain, CSF serves as a regulated conduit for growth factors, ions, extracellular vesicles, and other bioactive molecules. Emerging evidence suggests that the ChP contributes to shaping CSF composition through energy-dependent transport and state-responsive secretion. Ventricular-contacting NSCs sense CSF cues via apical endfeet and primary cilia, integrating signals to regulate their behavior. Lifespan-dependent remodeling of CSF composition and niche architecture reshapes NSC function from embryonic expansion to adult homeostasis and age-associated decline. Beyond the ventricular niche, ChP-derived factors influence circuit maturation and vulnerability to neurodegeneration. Orthodenticle homeobox 2 regulates critical period timing and neuroblast integration, whereas apolipoprotein E couples lipid metabolisms and amyloid-β homeostasis to neurogenesis with Alzheimer’s disease risk. Additional ChP-secreted proteins, including transthyretin and clusterin, further shape the extracellular proteostatic and lipid environment. Together, these findings support the view of the ChP-CSF axis as an adaptive regulator across the lifespan that integrates stem cell dynamics, circuit plasticity, and neurodegenerative susceptibility.

## Introduction

Cerebrospinal fluid (CSF) is a tightly regulated intracranial system that enables long-range signaling along and across the ventricular surfaces of the brain (Damkier et al., 2013). Beyond its protective role, CSF delivers growth factors essential for brain development and continues to operate throughout adulthood, maintaining ionic and neuroendocrine homeostasis while facilitating the metabolic waste clearance (Hladky and Barrand, 2016; Kaiser and Bryja, 2020). Because the composition of CSF defines the extracellular environment adjacent to the ventricles, even subtle changes can influence neural stem cell (NSC) behavior, neuronal excitability, and the neuro-supportive function of glial cells (Marques et al., 2007; Lehtinen et al., 2011; Lyckenvik et al., 2025). Most CSF is produced by the choroid plexus (ChP), a highly vascularized epithelial tissue within the brain ventricles (Brinker et al., 2014; Hladky and Barrand, 2014). The ChP acts both as a blood-CSF barrier and as a dynamic secretory organ, allowing systemic and brain-derived signals to rapidly reshape CSF composition (Saunders et al., 2023; Katada et al., 2025). This function is particularly relevant to ventricular-contacting NSCs, which directly interface with CSF through specialized apical endfeet and are therefore persistently exposed to ChP-derived signals (Silva-Vargas et al., 2013; Kaiser and Bryja, 2020). Although studies of the ChP–CSF axis have expanded rapidly (Saunders et al., 2023), key mechanistic questions remain. How do changes in CSF composition and dynamics fine-tune developmental processes, including neural circuitry formation? Which ChP programs orchestrate these shifts? Finally, how are these signals interpreted by ventricular-contacting NSCs to regulate quiescence, activation thresholds, long-term self-renewal, and the integration of newly generated neurons into functional neural circuits? In this mini-review, we summarize current evidence on ChP secretory mechanisms, how NSCs sense CSF cues, and the dynamic remodeling of ChP-CSF programs from development to aging, highlighting their roles in NSC maintenance and neural circuit formation.

## The choroid plexus as a dynamic secretory and transport hub

Cerebrospinal fluid is an actively maintained, high-turnover compartment that continuously distributes ions, proteins, and vesicular signals along the ventricular surfaces (Hladky and Barrand, 2016; Kaiser and Bryja, 2020). Approximately 70%–80% of CSF is produced by the ChP, with the remainder arising from extrachoroidal sources, including capillary/blood-brain barrier-associated pathways and interstitial fluid exchange (Brinker et al., 2014; Hladky and Barrand, 2014). Consistent with this role, the ChP is among the most highly perfused tissues in the brain (∼4 mL/min/g), exhibiting an approximately 10-fold greater perfusion rate than the brain parenchyma (Saunders et al., 2023). CSF production is not a passive ultrafiltration process but rather reflects an ATP-dependent, polarized transport program in which coordinated Na^+^, Cl^–^, and HCO^3–^ flux drives near-isotonic water movement into the ventricles (MacAulay et al., 2022). Although osmotic forces contribute, secretion can be sustained against opposing gradients, underscoring the primacy of active transport mechanisms (Oernbo et al., 2022).

Water flux involves aquaporin-1 (AQP1) as well as transporter-coupled and micro-osmotic processes. In addition, Na^+^-K^+^-2Cl^–^ cotransporter 1 (NKCC1) -dependent ion handling contributes to CSF K^+^ regulation and fluid homeostasis, particularly during early postnatal stages (Steffensen et al., 2018; Jensen et al., 2025). Importantly, CSF secretion is state-responsive. Neuromodulatory (Edelbo et al., 2025), endocrine (Nilsson et al., 1992), inflammatory (Tsitsou-Kampeli et al., 2024), and circadian inputs (Edelbo et al., 2023; Fame, 2025) dynamically reconfigure epithelial transport activity and barrier properties (Saunders et al., 2023). Beyond bulk fluid production, the ChP further refines CSF composition through merocrine protein release (Thouvenot et al., 2006), extracellular vesicle (EV), and apocrine secretion (Courtney and Lehtinen, 2025) providing additional temporal and spatial precision in signal delivery.

## Decoding CSF signals at the ventricular stem cell interface

One of the closest targets of the lateral ventricle ChP is the ventricular–subventricular zone (V-SVZ), where adult NSCs (B1 cells) contact the ventricle through specialized apical endfeet (Silva-Vargas et al., 2013). Accordingly, CSF transport dynamics and the mechanisms that allow CSF-derived signals to access ventricular-contacting stem cells are tightly regulated and critically influence stem cell behavior. Here, we highlight the cellular interfaces and signaling pathways that translate CSF exposure into stem cell state programs.

B1 cells integrate dual microenvironments, sampling CSF through a restricted apical microdomain while extending a basal process to contact blood vessels (Shen et al., 2008; Llorente et al., 2022). Multiciliate ependymal cells (E cells) organize around B1 apical endings to form “pinwheel” structures that compartmentalize and buffer CSF exposure (Mirzadeh et al., 2008; Llorente et al., 2022). At the apical face, B1 cells extend a primary cilium into the ventricular space, where Hedgehog components are enriched, enabling efficient detection of CSF-borne ligands such as Sonic hedgehog and activation of Gli-dependent transcription (Tong et al., 2014). Adhesion molecules, including vascular cell adhesion molecule 1 (VCAM1), further stabilize this architecture and couple inflammatory cues to stem cell quiescence (Kokovay et al., 2012).

Beyond structural specification, B1 cells decode CSF signals through ligand–receptor signaling. Within the ChP–CSF–NSC axis, ChP-derived insulin-like growth factor 2 (IGF2) activates AKT and ERK signaling in an stage-dependent manner (Lehtinen et al., 2011). CSF flow itself also acts as a physical cue. Planar-polarized ependymal cilia generate a directional near-wall flow that shapes local concentration gradients of guidance molecules, thereby influencing SVZ outputs such as neuroblast migration (Siyahhan et al., 2014). In parallel, ventricular-contacting NSCs can directly sense flow via epithelial sodium channels (ENaC), where Na^+^ influx triggers Ca^2+^ signaling and ERK activation to promote NSC proliferation (Petrik et al., 2018). More recently, ependymal ciliary beating has been suggested to maintain NSC quiescence by generating mechanosensitive Ca^2+^ transients through the PKD1/2–TRPM3 pathway, representing a second mechanism by which CSF flow regulates stem cell state, distinct from ENaC signaling (Bressan et al., 2026).

In addition to rapid signaling events, NSCs internalize EVs, enabling slower transcriptional reprogramming through microRNA cargo (Lepko et al., 2019; Liu et al., 2025). Although heparan sulfate proteoglycans (HSPGs) contribute to EV uptake in diverse systems and HSPG-rich extracellular matrix specializations in the niche may enhance EV retention, how NSCs internalize EVs remains poorly understood (Christianson et al., 2013). Importantly, CSF signaling is likely state-dependent, however, our understanding of how endocrine status, circadian phase, and sex influence these processes remains limited.

## Lifespan remodeling of the ChP-CSF axis and stem cell niche dynamics

Cerebrospinal fluid composition and flow dynamics evolve across maturation and aging, and the ventricular wall itself undergoes structural remodeling that alters how NSCs sample ventricular cues. Understanding how the ChP-CSF axis supports V-SVZ pool maintenance from embryogenesis to old age requires clarifying how age-dependent changes in ChP output and ventricular exposure are translated into niche programs.

**FIGURE 1:**
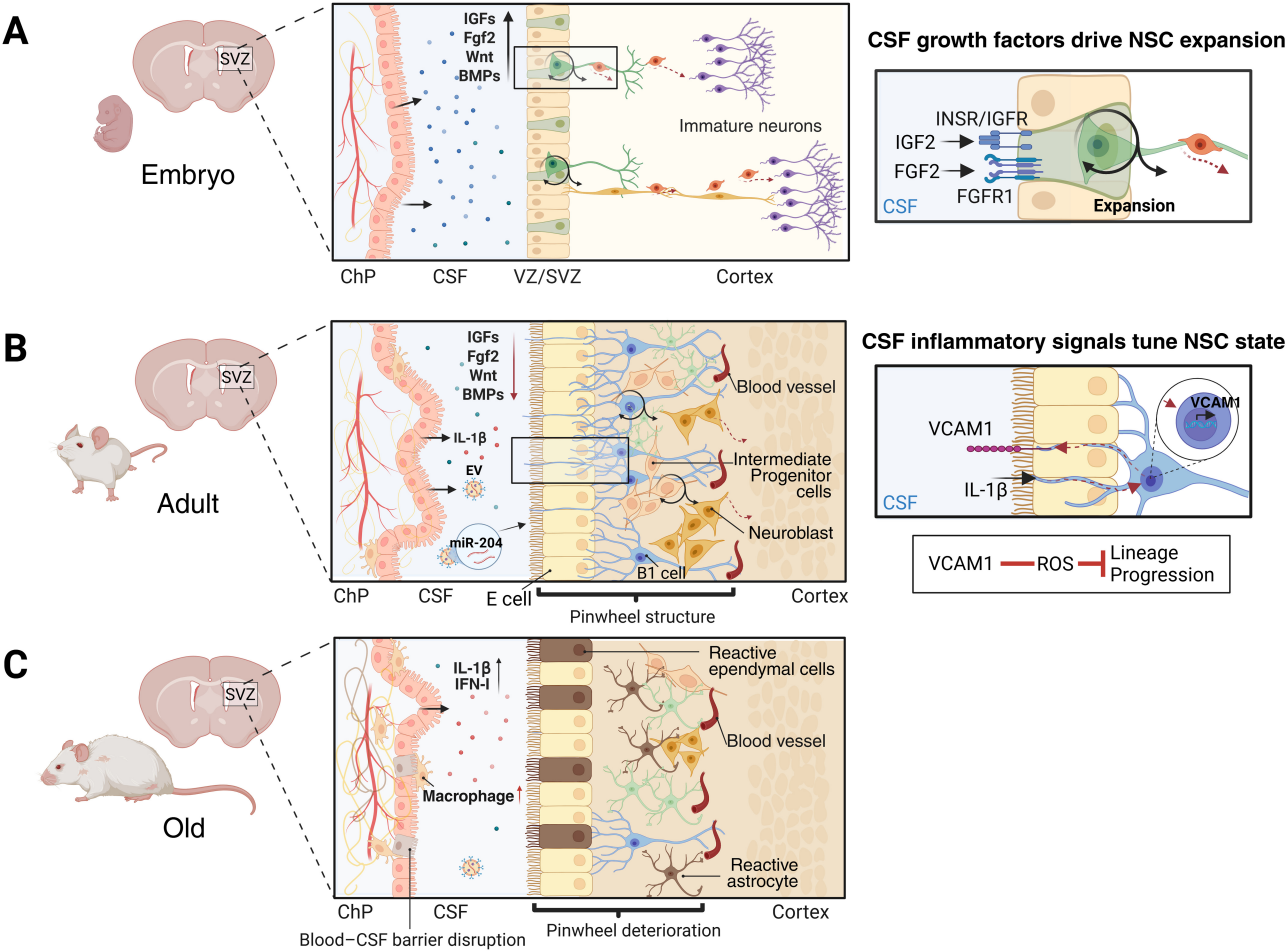
Three labeled panels illustrate changes in cerebrospinal fluid regulation of neural stem cells in the ventricular-subventricular zone (V-SVZ) across life stages in mice. **(A)** Depicts the embryo with growth factors in CSF promoting neural stem cell expansion. **(B)** Shows the adult with both growth and inflammatory signals in CSF influencing niche state, including VCAM1 and IL-1β. **(C)** Represents old age, highlighting blood-CSF barrier disruption, elevated inflammatory cytokines, macrophage presence, and reactive cell types, indicating diminished regulation and increased inflammation. Each panel includes detailed diagrams of mouse brain regions, cellular interactions, and key molecular pathways at each stage.

During embryogenesis, ventricular NSCs are primarily engaged in rapid pool expansion and fate specification and CSF functions as a critical trophic and patterning milieu (Figure 1A). Early experimental studies demonstrated that CSF-derived factors are required for neuroepithelial survival and proliferation (Gato et al., 2005). Subsequent work identified the ChP as a central source of developmental signals within the CSF, including mitogenic cues such as IGF2 and FGF2 that promote progenitor expansion through receptor activation at the primary cilium of ventricular NSCs (Figure 1A; Martín et al., 2006; Lehtinen et al., 2011). Transcriptomic and proteomic profiling further revealed that the developing ChP expresses coordinated signaling modules, including WNTs, BMPs, and retinoic acid metabolic enzymes, consistent with a role in shaping regional identity and growth programs of the embryonic brain (Lun et al., 2015; Dani et al., 2021). Emerging evidence also suggests that embryonic ChP-CSF interface serves a protective function by limiting inflammatory exposure in the immature brain (Kudo et al., 2026). Together, these findings position the embryonic ChP-CSF axis as a developmentally tuned signaling hub that integrates trophic support, patterning information and barrier protection to sustain neuroepithelial expansion.

In adulthood, the system shifts from expansion to fine control of quiescence and activation (Figure 1B). VCAM1-mediated adhesion reinforces pinwheel organization and long-term quiescence. CSF-borne IL-1β upregulates VCAM1 on B1 apical endfeet and engages VCAM1–NOX2/ROS signaling to retains inappropriate activation under inflammatory conditions (Figure 1B; Kokovay et al., 2012). Ventricular flow and ependymal cilia provide additional instructive cues, as ventricular-contacting NSCs convert flow into proliferative signaling (Sawamoto et al., 2006; Petrik et al., 2018). Slower gene-regulatory control is mediated by ChP-derived cargos such as CSF miR-204, which derived from ChP helps maintain the quiescent NSC pool (Lepko et al., 2019). IGF2 signaling also remains relevant in adulthood (Ziegler et al., 2019), supporting long-term label-retaining stem cells. In particular, the insulin receptor (INSR) is required for self-renewal of specific SVZ NSC subsets, consistent with an IGF2-INSR-dependent homeostatic circuit (Chidambaram et al., 2022).

With aging, the ventricular interface is progressively remodeled: pinwheel microdomains decline (Shook et al., 2012), niche astrocytes and ependymal cells acquire reactive-like features, and B1 proliferation decreases (Figure 1C; Spassky et al., 2005; Capilla-Gonzalez et al., 2014). Concurrently, the ChP undergoes molecular reprogramming with reduced expression of neurotrophic and homeostatic factors (e.g., BDNF, IGF1/IGF2) and alterations in barrier components (Sadanandan et al., 2024). Beyond trophic depletion, aging drives an inflammatory and host-defense reprogramming of the ChP, marked by a persistent type I interferon (IFN-I) signature and increased IL-1β signaling associated with macrophages recruitment (Baruch et al., 2014; Dani et al., 2021). Consistent with increased macrophage presence, aging-associated senescence in the ChP leads to hyperactivation of macrophage-derived cathepsin S (CTSS) in the stromal compartment in the ChP. Then CTSS cleaves the tight-junction component claudin-1 (CLDN1) in ChP epithelial cells, thereby compromising the blood–CSF barrier (Chen et al., 2025). Importantly, inhibition of CTSS restores CLDN1 and tight-junction integrity, reduces inflammatory readouts (including TNF-α in the SVZ and hippocampus), and rescues both SVZ cell proliferation and behavior deficits in aged mice (Chen et al., 2025).

Notably, aged V-SVZ cells retain responsiveness to soluble factors enriched in young CSF, particularly IGF1 and BMP5), as exposure to young CSF enhances proliferation and partially restores differentiation capacity (Silva-Vargas et al., 2016). A similar phenomenon is observed at the systemic level, where exposure to young circulation via heterochronic parabiosis enhances adult neurogenesis (Villeda et al., 2011), and young blood can reverse age-related deficits in cognitive function and synaptic plasticity (Villeda et al., 2014). Together, these findings support the idea that systemic circulating signals converge on CSF-accessible interfaces to reshape neurogenic output. However, a key unresolved question is whether age-related SVZ decline is driven primarily by changes in the ChP-CSF axis or by remodeling within the niche itself. On one hand, disruption of the ChP barrier can act as a causal mechanism, as it is sufficient to elevate inflammatory tone and suppress SVZ proliferation. On the other hand, niche-intrinsic mechanisms independent of the ChP-CSF axis also contribute, as ageing increases vascular-derived TGF-β signaling, which acts as an anti-proliferative factor in the SVZ (Pineda et al., 2013).

## Beyond the ventricle: ChP-CSF modulates neural plasticity and neurodegeneration

Importantly, the influence of the ChP-CSF axis extends beyond ventricular-contacting stem cells to distant brain regions, where it shapes circuit maturation and experience-dependent plasticity.

**FIGURE 2:**
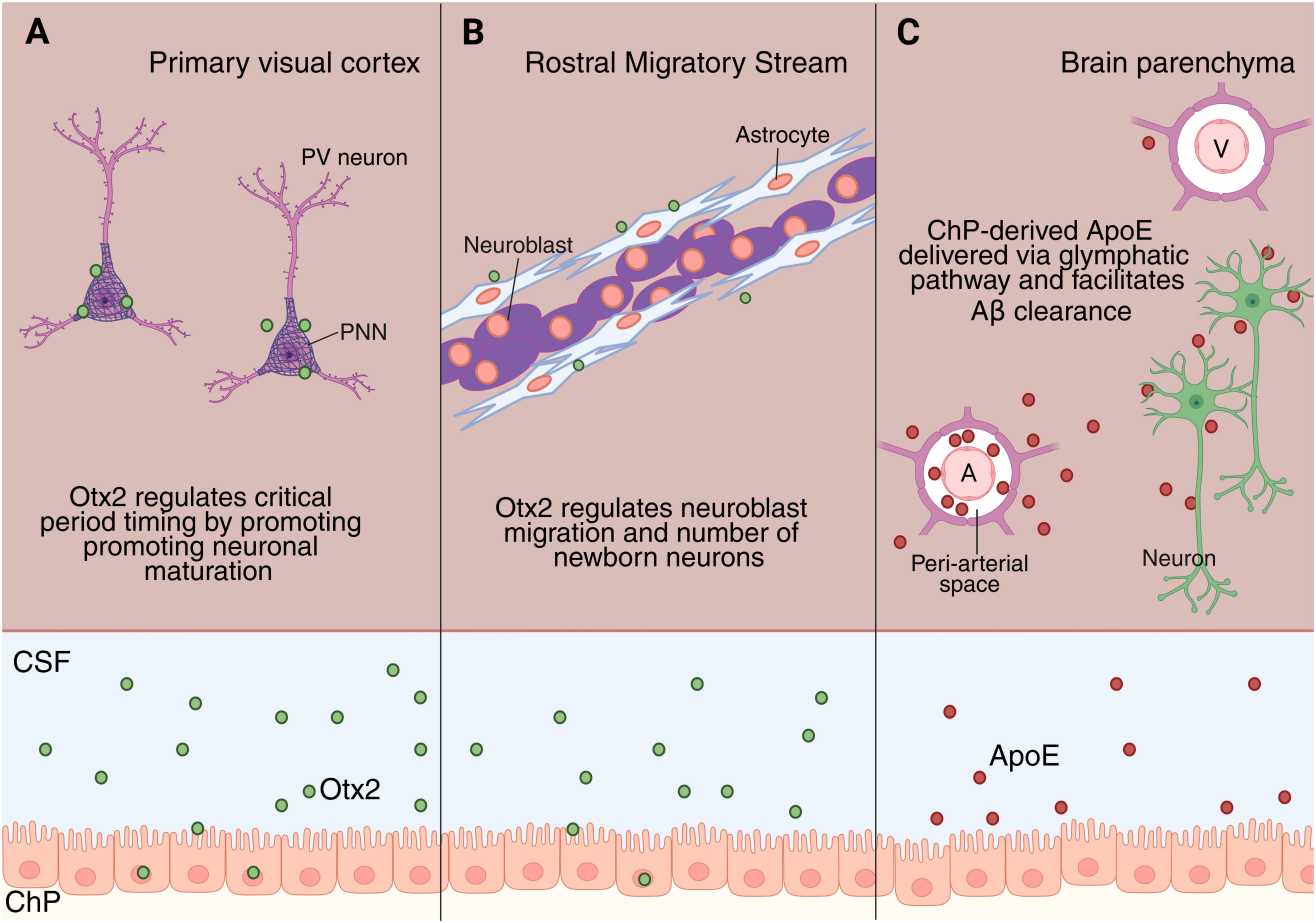
Choroid plexus-cerebrospinal fluid (ChP-CSF) axis modulates brain function beyond the ventricle. **(A)** Shows Otx2 transport from ChP through CSF to PV neurons in the primary visual cortex, promoting neuronal maturation. **(B)** Depicts Otx2 in the rostral migratory stream, regulating neuroblast migration and number of newborn neurons. **(C)** Illustrates ApoE produced by ChP, transported into the brain along periarterial (A) CSF influx and cleared via perivenous (V) pathways to the venous circulation.

One prominent example is orthodenticle homeobox 2 (*Otx2*), a homeobox transcription factor essential for ChP development and function (Johansson et al., 2013). Beyond this intrinsic role, OTX2 is secreted into the CSF and acts non-cell-autonomously to regulate postnatal neuroplasticity and neurogenesis. During the postnatal critical period, a defined developmental window in which sensory experience refines neural circuits, ocular dominance plasticity, a key mechanism underlying binocular vision, is shaped by visual input (Wiesel and Hubel, 1963). In this context, accumulation of OTX2 in the primary visual cortex plays a pivotal role in determining its timing (Figure 2A). Sugiyama et al. (2008) demonstrated that cortical infusion of OTX2 accelerated critical period onset, whereas *Otx2* reduction delays the parvalbumin (PV) interneuron maturation and prolongs plasticity. Notably, PV interneurons do not express *Otx2* themselves (Sugiyama et al., 2008). Subsequent studies identified the ChP as a major source of CSF-borne OTX2 (Spatazza et al., 2013). After secretion into the CSF, OTX2 is preferentially internalized by PV interneurons via the perineuronal nets in the primary visual cortex, promoting their maturation and driving critical period closure (Figure 2A). Importantly, reducing *Otx2* expression specifically in the ChP reopens cortical plasticity in adult mice (Spatazza et al., 2013). These findings support the two-threshold model, in which distinct levels of OTX2 accumulation switch plasticity on and off, directly connecting the ChP secretory state to the temporal regulation of brain circuits.

OTX2 has also been shown to regulate plasticity in the primary auditory (A1) and medial prefrontal cortex (mPFC) by promoting PV interneuron maturation, supporting tonotopic map expansion in A1 and acoustic preference learning in the mPFC, respectively (Lee et al., 2017). More recently, non-cell-autonomous OTX2 signaling in the piriform cortex has been shown to similarly control PV interneuron maturation states and olfactory-driven behavior (Gibel-Russo and Di Nardo, 2024). Together, these findings reveal that OTX2 signaling exerts widespread effects across the postnatal cortex, shaping maturational trajectories across distinct sensory modalities, including visual, auditory, and olfactory systems. Although it is unclear whether these effects are mediated by ChP-derived OTX2, its widespread non-cell autonomous distribution in the adult cortex and the fact that the ChP is a major source of OTX2 suggests that the ChP may broadly contribute to neural plasticity.

Beyond cortical plasticity, ChP-derived OTX2 also regulates neuroblast migration from the V-SVZ to the olfactory bulb (Figure 2B). Either reducing Otx2 expression in the ChP or sequestering OTX2 within the CSF decreases the number of newborn neurons reaching the olfactory bulb by altering extracellular matrix components and signaling molecules in supporting astrocytes, without affecting V-SVZ cell proliferation (Planques et al., 2019), highlighting its selective role in postnatal circuit integration rather than stem cell expansion.

In addition to OTX2, the ChP-CSF axis may influence brain aging and neurodegenerative disease through apolipoprotein E (ApoE). ApoE plays a central role in amyloid-β clearance, a hallmark of Alzheimer’s disease (Jiang et al., 2008), and exists as three major isoforms (ApoE2, ApoE3, ApoE4), with ApoE4 representing the strongest genetic risk factor (Corder et al., 1994). While ApoE in the brain parenchyma is predominantly produced by astrocytes, the ChP also expresses high levels of ApoE and constitutes a major source of CSF-borne ApoE (Xu et al., 2006). Recent studies have reported that ChP-derived ApoE can delivered into the brain parenchyma through perivascular and glymphatic pathways in an isoform-dependent manner (ApoE2 > ApoE3 > ApoE4) (Achariyar et al., 2016), thereby potentially shaping regional amyloid deposition and neuronal circuit vulnerability (Figure 2C).

Beyond its role in amyloid metabolism, ApoE also contributes to neurogenesis and synaptic plasticity (Mauch et al., 2001; Li et al., 2009; Yang et al., 2011). Experimental studies indicate that ApoE signaling influences NSC proliferation, neuronal maturation, and dendritic complexity, suggesting that CSF-borne ApoE may link stem cell regulation with circuit remodeling. In this context, additional ChP-enriched proteins may cooperate in shaping the extracellular environment. Transthyretin (TTR), a thyroid hormone-binding protein secreted abundantly by the ChP, interacts with amyloid-β and has been implicated in neuroprotection (Silva et al., 2017). Reduced TTR levels have been associated with aging and Alzheimer’s disease, raising the possibility that ChP-derived TTR contributes to maintaining proteostatic balance in the CSF (Serot et al., 1997). Clusterin (ApoJ), another CSF-enriched apolipoprotein associated with Alzheimer’s disease risk, participates in lipid transport, complement regulation, and amyloid handling (Miners et al., 2017; Ko et al., 2022). Together, these findings suggest that the ChP-CSF axis regulates not only stem cell dynamics and circuit plasticity but also the extracellular proteostatic and lipid landscape that influence susceptibility to neurodegenerative disease.

## Perspectives

Emerging evidence suggests that the ChP-CSF axis is a dynamic regulator of both NSC behavior and circuit function across the lifespan. Rather than serving solely as barrier or CSF-producing epithelium, the ChP releases signaling molecules into the CSF that influence ventricular niche organization, neuroblast migration and their subsequent integration into functional circuits.

This integrated view raises important questions. How systemic physiological factors such as nutritional state, circadian timing, hormonal regulation, and sex differences further refine ChP secretory programs and CSF-mediated signaling. A growing number of recent studies have begun to highlight these dimensions (Fame et al., 2023; Kaiser et al., 2025), suggesting that metabolic status, endocrine cues, and daily rhythms can modulate CSF composition and niche responsiveness in ways that impact both NSC behavior and circuit function.

A deeper understanding of these mechanisms may redefine the ChP-CSF axis as an adaptive interface linking peripheral physiology to central plasticity. Notably, the preserved responsiveness of aged NSCs to youthful CSF suggests that aspects of niche decline may be reversible. Targeting ChP secretory programs or restoring CSF signaling balance could therefore represent an upstream strategy to simultaneously modulate neurogenesis, circuit plasticity, and vulnerability to neurodegeneration.

Addressing these challenges will require integrating stem cell biology, circuit-level functional analyses, and high-resolution molecular profiling of ChP and CSF dynamics. Elucidating how the ChP-CSF axis synchronizes stem cell states with circuit demands may uncover a unifying framework connecting brain development, plasticity and degeneration.
